# A 6-Week Program to Strengthen Resiliency Among Women With Metastatic Cancer: A Randomized Clinical Trial

**DOI:** 10.1093/oncolo/oyad091

**Published:** 2023-04-27

**Authors:** Arash Asher, Celina H Shirazipour, Jessica M Capaldi, Sungjin Kim, Marcio Diniz, Bronwen Jones, Jeffrey Wertheimer

**Affiliations:** Department of Medicine and Physical Medicine and Rehabilitation and Patient and Family Support Program at Cedars-Sinai Cancer, Los Angeles, CA, USA; Division of Population Sciences, Cedars-Sinai Cancer, Los Angeles, CA, USA; David Geffen School of Medicine, University of California Los Angeles, Los Angeles, CA, USA; Division of Population Sciences, Cedars-Sinai Cancer, Los Angeles, CA, USA; Biostatistics Research Center, Samuel Oschin Comprehensive Cancer Center and Departments of Medicine and Biomedical Sciences, Los Angeles, CA, USA; Biostatistics Research Center, Samuel Oschin Comprehensive Cancer Center and Departments of Medicine and Biomedical Sciences, Los Angeles, CA, USA; Department of Spiritual Care, Cedars-Sinai Cancer, Los Angeles, CA, USA; Department of Physical Medicine and Rehabilitation at Cedars-Sinai Medical Center, Los Angeles, CA, USA

**Keywords:** spirituality, qualify of life, health promotion, breast cancer

## Abstract

**Purpose:**

The objective of this study was to evaluate the effect of an intervention (Growing Resilience And CouragE; GRACE) on spiritual well-being, quality of life, and general well-being in women with metastatic cancers reporting existential or spiritual distress.

**Patients and Methods:**

Prospective, randomized, wait-list control clinical trial. Women with metastatic cancer experiencing existential or spiritual concerns were randomized to GRACE or waitlist control. Survey data were collected at baseline, end of program, and 1-month follow-up. Participants included English-speaking women, 18 or older, with metastatic cancer, existential or spiritual concerns, and reasonable medical stability. Eighty-one women were assessed for eligibility; 10 were excluded (not meeting exclusion criteria, refusal to participate, and death). The primary outcome was spiritual well-being measured pre- and post-program. Secondary measures assessed quality of life, anxiety, depression, hopelessness, and loneliness.

**Results:**

Seventy-one women (aged 47-72) were enrolled (GRACE *n* = 37, waitlist control *n* = 34). GRACE participants demonstrated significant improvements in spiritual well-being compared to control at end of program (parameter estimate (PE), 16.67, 95% CI, 13.17, 20.16) and 1-month follow-up (PE, 10.31, 95% CI, 6.73, 13.89). Additionally, significant improvements were demonstrated in quality of life at the end of program (PE, 8.51, 95% CI, 4.26, 12.76) and 1-month follow-up (PE, 6.17, 95% CI, 1.75, 10.58). GRACE participants also demonstrated improved depression and hopelessness at follow-up, as well as improved anxiety.

**Conclusions:**

Findings suggest the value of evidence-based psychoeducational and experiential interventions for improving the well-being and quality of life of women with advanced cancer.

**Trial Registration:**

ClinicalTrials.gov Identifier: NCT02707510.

## Teaser

Growing resilience and courage (GRACE) is a 6-week program developed to improve spiritual well-being, quality of life, and general well-being of women with metastatic cancer reporting spiritual or existential distress. This article reports the results of a study that evaluated the effect of the intervention.

**Implications for Practice**

Growing Resilience And CouragE (GRACE) is a 6-week program developed to improve spiritual well-being, quality of life, and general well-being among women with metastatic cancer reporting spiritual or existential distress. In this randomized clinical trial, participants in GRACE demonstrated significant improvements in spiritual well-being, quality of life, depression, and hopelessness compared to waitlist control through 1-month post-intervention. Intervention participants also demonstrated greater improvements in anxiety. Individuals with advanced cancer experiencing profound existential and spiritual challenges can demonstrate improvements in well-being and quality of life through participation in a multi-faceted, psycho-educational program.

## Introduction

Women with metastatic breast cancer now have a meaningful improvement in survival, with a median overall survival of about 2 years.^1^ Some women live many years with metastatic breast cancer^2,3^ thanks to the availability of newer systemic therapies. Survival, however, is not on its own an adequate measure of success when viewed from the lens of the biopsychosocial model.^4^ Paradoxically, the success of new treatments and subsequent increases in survival brings to the forefront the potential for existential and spiritual distress,^5^ which may include feelings of isolation, alienation, meaninglessness, and a prodigious sense of angst. As women live longer with a diagnosis of metastatic cancer, there is the potential for greater open awareness of and preoccupation with the nearness of dying.^6^

The quality-of-life continuum has been described as a dialectic that extends from suffering and anguish at one extreme to an experience of integrity and wholeness at the other.^7^ Patients with advanced cancer often face demoralization and profound existential and spiritual challenges that can adversely impact qualify of life and medical outcomes.^8-10^ Although existential and spiritual distress—which can include transpersonal, interpersonal, and intrapersonal dimensions—is one of the most debilitating conditions experienced by patients with advanced cancer, it is often a neglected area of cancer care.^11,12^ The National Comprehensive Cancer Network (NCCN) guidelines in 1997 thus focused specifically on this concept of distress, defining it as a “multifactorial unpleasant experience of psychological (eg, cognitive, behavioral, and emotional), social, spiritual, and/or physical nature that may interfere with the ability to cope effectively with cancer, its physical symptoms and its treatment.”^13^ Distress was further defined as a continuum of emotions with normal feelings of sadness on one end through to debilitating experiences including existential and spiritual crisis.^13^ Through this lens, a life-threatening illness such as metastatic cancer can be interpreted as an assault on the whole person that may bring existential suffering to patients as an inevitable consequence of the disease and treatment.^5^ As a result, spiritual care has been a part of the NCCN guidelines for Distress management since 1997.^13^

Prevalence rates for elevated levels of psychological distress are significant in individuals with advanced cancer. Plumb and Holland^14^ reported that 20%-30% of patients admitted to the hospital for treatment of advanced cancer developed clinically significant depression, and 15% had severe anxiety. In a study of 215 individuals with cancer, 47% of the patients received a psychiatric diagnosis^15^; of those participants with psychiatric disturbance, approximately 68% of the psychiatric diagnoses consisted of adjustment disorders, with 13% representing major affective disorders (depression).^15^ Multiple reports cite the incidence of anxiety in advanced cancer patients to be between 6% and 34%,^16-21^ with at least one study citing up to 49%.^22^ Furthermore, Delgado-Guay et al^23^ found that increased spiritual or existential pain correlated with depression, anxiety, pain, and general well-being. Suffice it to say, being confronted with a progressive medical condition can ensue in psychological distress for many and will impact the experience of “living” for all. Considering issues related to quality of life is crucial in this context.

GRACE (Growing Resilience And CouragE) is a 6-week multi-faced, psycho-educational program developed with the goal of enabling individuals with cancer to find viable solutions to move toward an experience of integrity and wholeness. Meaning-centered psychotherapeutic individual and group intervention has been found to have successful outcomes in the cancer population.^24-27^ A limitation of previous existential interventions was a lack of theoretical foundation for the intervention.^28^ GRACE addresses these limitations and is guided by 4 evidence-based approaches to improving quality of life and resiliency in the cancer setting: (1) logotherapy (existential therapy)^29^; (2) cognitive-behavioral therapy^30^; (3) mindfulness^31^; and (4) positive psychology.^32^A retrospective study of GRACE demonstrated that participation significantly improved depression, anxiety, hopelessness, and spiritual well-being among 42 patients with metastatic cancer and subjective existential concerns.^33^ These findings led to the current study: a prospective, randomized clinical trial (RCT) designed to assess the impact of the GRACE program on spiritual well-being, quality of life, and general well-being among women with metastatic cancer reporting spiritual or existential distress.

## Materials and Methods

### Design

**Figure 1.**
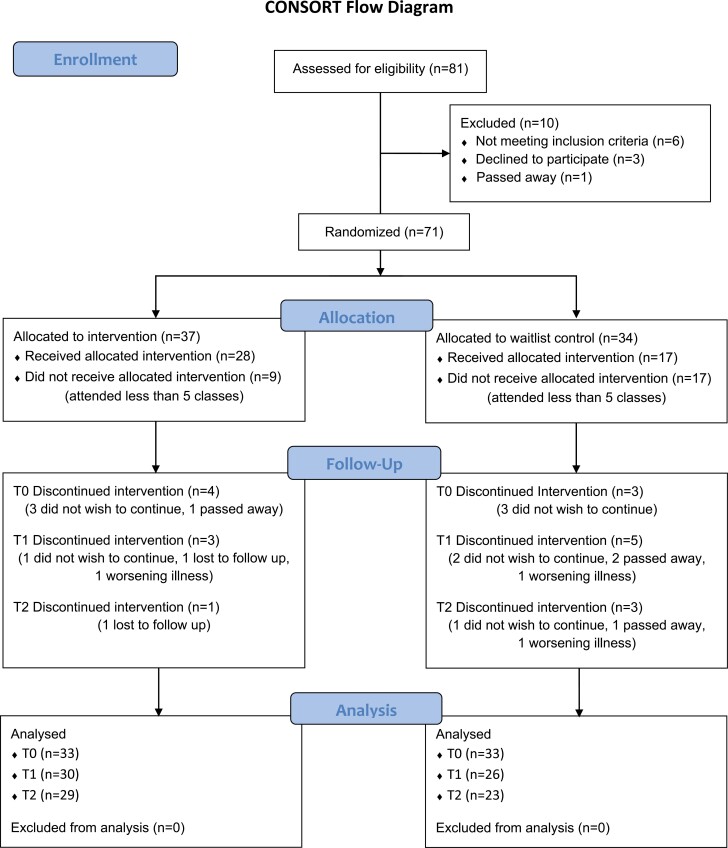
CONSORT diagram.

The study is a non-blinded, randomized, wait-list control clinical trial. Study approval was provided by the *removed for blind review* institutional review board. Participants were recruited via referral and word of mouth at CSMC and its affiliates between October 2016 and March 2019. The study’s clinical research specialist enrolled and randomized patients in a block balanced fashion using computer-generated random assignment 2 weeks before the start of GRACE. Each participant signed an informed consent document in accordance with institutional and national guidelines. This study followed the Consolidated Standards of Reporting Trials reporting guideline (Fig. 1).

#### Intervention

Participants in the intervention group were provided with all programmatic materials (including copies of power point presentations, copies of reading texts, and audio CDs) and attended set-time weekly group classes over 6-weeks facilitated jointly by the principal investigators (XX, XX) and the cancer center chaplain (XX). All intervention participants completed surveys before participating in GRACE (T0), immediately after GRACE (T1), and 1 month after GRACE (T2).

#### Waitlist Control

Participants in the control group completed surveys at baseline (T0), 6 weeks after baseline (T1), and 1 month after T1 (T2). After completion of the waitlist period, they had the opportunity to participate in GRACE.

### Participants

We enrolled English-speaking clinic patients 18 years and older, with a diagnosis of metastatic cancer (minimum prognosis of 3 months or more), existential or spiritual concerns (based on screening survey), and reasonable medical stability as assessed by the evaluating physician. Exclusion criteria included unstable psychiatric disorders that would detract from a group program (eg, uncontrolled depression/anxiety, volatile personality disorders). Women with brain metastasis with significant cognitive impairment that would preclude participation in a psycho-education program or cognitive linguistic impairment (eg, aphasia) were also excluded.

### Intervention

GRACE incorporates both psycho-educational and experiential interventions. The curriculum is standardized and includes themes illustrated via PowerPoint slides with structured delivery, video presentations, a variety of mindfulness meditation practices, and selected readings that serve to reflect and capture the theme for the week of the curriculum, with a substantial emphasis on Dr Rachel Naomi Remen’s, *My Grandfather’s Blessings* and Dr Viktor E. Frankl’s *Man’s Search for Meaning*. Homework assignments, or reflective opportunities, were included in each session to help reinforce key ideas and concepts.

GRACE was designed to provide a sense of awareness and control, develop ways of finding meaning and purpose, learn skills to enhance perspective management, strengthen connectivity, develop a sense of gratitude despite difficult circumstances, identify and utilize personal strengths and virtues, and to help crystallize one’s legacy as a means to living the most purposeful life possible. In essence, GRACE was designed to provide a structured, systematic way of building coping and resiliency skills under difficult life circumstances to address a major gap in comprehensive cancer care.

### Objectives

This RCT sought to examine the effect of an empirically anchored 6-week psycho-educational program on spiritual well-being, quality of life, and general well-being compared to a waitlist control. The primary objective was to quantify the impact of the GRACE program on metastatic cancer participants’ sense of spiritual well-being. Secondary objectives included assessments of quality of life, and general well-being (anxiety, depression, loneliness, and hopelessness).

### Outcome Measures

Attendance and attrition information, demographic characteristics, and cancer information were collected. Participants also completed 6 validated measures of spiritual well-being, quality of life, and general well-being at all timepoints.

#### Attendance and Attrition

Attendance information was collected for all sessions. Compliance was defined as attending at least 5 out of the 6 sessions. If participants left the study, they were asked to provide a reason to the research coordinator.

#### Demographic Characteristics and Medical Information

Demographic characteristics and medical information were collected through patient medical records and supplemented with surveys. Demographic information collected included age, race, ethnicity, marital status, household composition, education, previous treatment with mental health professionals, and primary language. Medical information collected included location of metastasis, treatment type, timeline of active treatment, months since initial positive diagnosis, months since initial metastatic disease diagnosis, and history of depression, anxiety, bipolar disorder, or other psychiatric disorder.

#### Spiritual Well-Being

To assess spiritual well-being, participants completed the Functional Assessment of Chronic Illness Therapy (FACIT) Spiritual Well-Being Scale—Expanded (FACIT-Sp).^34-36^ The FACIT-Sp is a reliable and validated 12-item self-administered questionnaire.^34^ The questionnaire assesses 2 domains of spiritual well-being: meaning/peace and faith. Higher scores indicate a higher level of spiritual well-being.

#### General Quality of Life

Participants completed the Functional Assessment of Cancer Therapy-General (FACT-G) to assess general quality of life.^37^ This is a 27-item scale demonstrating strong reliability and validity in cancer populations that assesses quality of life and well-being across 4 domains: physical (eg, pain); social/family (eg, support); emotional (eg, fears around illness); and functional well-being (eg, ability to work).^38-40^ Higher scores indicate greater quality of life.

#### General Well-being

General well-being was assessed through 4 different surveys measuring anxiety, depression, hopelessness, and loneliness.

##### Anxiety

Anxiety was assessed using the Beck Anxiety Inventory (BAI)^41^ This 21-item survey measures common symptoms of anxiety over the previous week, including psychological (eg, fear of the worst happening), and somatic symptoms (eg, heart pounding or racing). This survey has demonstrated strong reliability and validity.^42^ For this research, in addition to a total score, 2 additional components were used to differentiate “cognitive” or “psychic anxiety” (emotional distress) and “somatic anxiety” (physical symptoms). Higher scores indicate greater anxiety.

##### Depression

Depression was assessed using the 7-item Beck Depression Inventory-Fast Screen (BDI-FS),^43^ which screens for severity of depression in adolescents and adults over the previous 2 weeks. The BDI-FS focuses on psychological symptoms corresponding to the non-somatic criteria of depression, including hopelessness, past failure, loss of pleasure, and suicidal ideation. This survey demonstrates strong reliability and validity.^44,45^ Higher scores indicate more severe depression.

##### Hopelessness

Hopelessness was measured using the 20-item Beck Hopelessness Scale (BHS)^.46^ This scale assesses 3 domains of hopelessness: feelings about the future, loss of motivation, and expectations. This survey demonstrates strong reliability and validity.^47-49^ Higher scores indicate greater hopelessness.

##### Loneliness

Loneliness was measured using the 20-item UCLA Loneliness Scale Version 3.^50^ The survey assesses subjective feelings of loneliness and isolation. This survey demonstrates strong reliability and validity.^50,51^ Higher scores reflect greater loneliness.

### Statistical Methods

#### Sample Size Calculations

Statistical power was assessed to detect the difference in change of the psychometric measures from baseline to post-intervention between GRACE participants and the waitlist control group using the 2-sided, 2-sample *t*-test assuming equal variance at the significance level of 0.05 and 80% power. Based on pilot data and anticipated attrition, the estimated sample size was 60 evaluable participants.

#### Analysis

**Figure 2.**
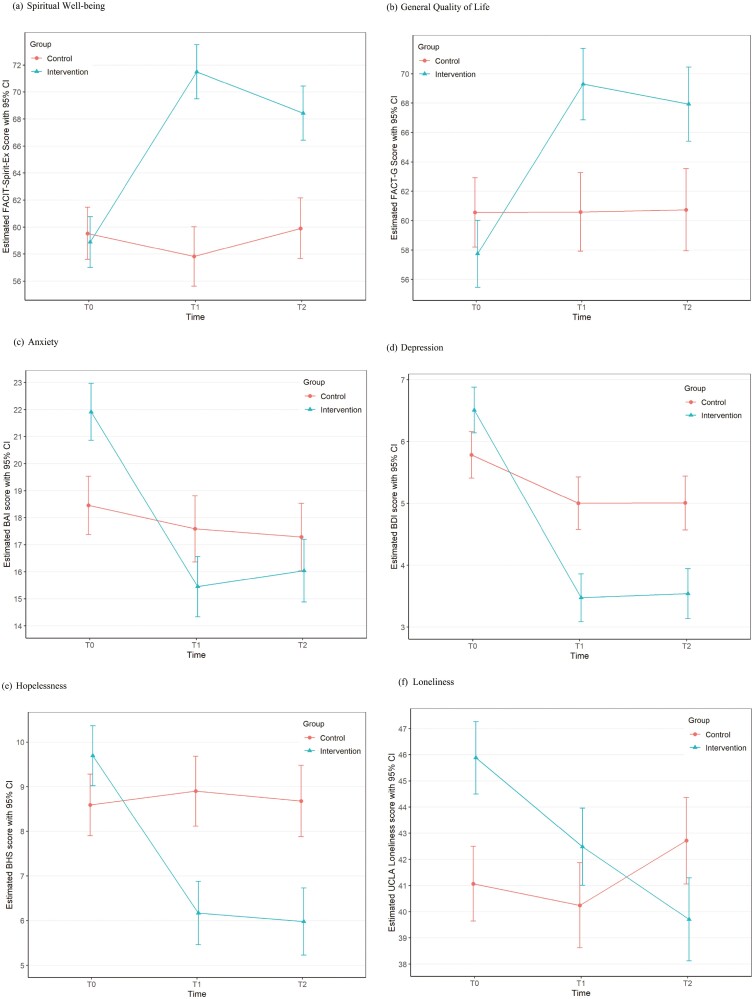
Outcomes for each group over time. For all figures*: Note.* Unadjusted estimated scores.

Patient characteristics, attrition rate, and compliance rate (defined as attending at least 5 out of 6 classes) were compared between intervention and control groups using Wilcoxon rank-sum test for continuous variables and chi-square test or Fisher’s exact test for categorical variables as appropriate. Baseline assessments occurred before randomization, and clinical characteristics pertaining to psychological status could not be controlled. Individual outcomes assessed at T0, T1, and T2 were modeled to examine if there is a GRACE program effect (intervention vs. control) on each outcome over time using a generalized additive model for location, scale, and shape (GAMLSS)^52^ with patient as a random effect and including an interaction term between group (intervention vs. control) and time with and without adjustment for covariates. A normal distribution was used to model FACIT-Sp score, FACT-G score, and UCLA Loneliness score; and a Gumbel distribution with an identity link function was used to model BAI score, BDI-FS score, and BHS score. Unadjusted estimated value of each outcome for each group at each timepoint is graphically presented (Fig. 2). The goodness of fit of each model was examined using residuals and the generalized Akaike information criterion (GAIC) such that the most adequate response distribution is chosen.^53^ Model selection was performed using a stepwise variable selection procedure based on GAIC. All analyses were performed using R package version 4.0.5^54^ with 2-sided tests at a significant level of 0.05.

## Results

### Patient Accrual and Study Compliance

A total of 71 participants were enrolled and randomized to the study. Baseline patient characteristics and clinical data were balanced between the study arms (Tables 1 and 2), except for primary language spoken. The percentage of participants who attended 5 of 6 classes was significantly higher (*P* = .025) in the intervention group (76% or 28 out of 37 participants) compared to the control group (50% or 17 out of 34 participants). Of note, 16 (43.24%) participants in the intervention group completed all sessions, compared to 13 (38.24%) in the control group. There was no significant difference (*P* = .308) in attrition rates between the intervention (21.62%) and control (32.35%) groups. The most common reason for withdrawal from the study included voluntary withdrawal after enrollment [*n* = 10; *n* = 4 (6%) intervention, *n* = 6 (8%) control group]. Other reasons for withdrawal included death (*n* = 4), worsening illness (*n* = 3), and loss of follow-up (*n* = 2) (Fig. 1).

**Table 1.** Participant demographic characteristics and medical information, stratified by group.

Variable	All patients (*N* = 64)	Intervention (*N* = 33)	Control (*N* = 31)	*P*-value
Participant demographic characteristics Age at class (Years)				
Median (IQR)	61 (47—72)	63.5 (54.5—73)	59.5 (40—71)	.197
Race				
White	51 (86.44)	26 (89.66)	25 (83.33)	1.000
Asian	3 (5.08)	1 (3.45)	2 (6.67)	
Black or African American	2 (3.39)	1 (3.45)	1 (3.33)	
Unknown/not reported	2 (3.39)	1 (3.45)	1 (3.33)	
More than one race	1 (1.69)	0 (0)	1 (3.33)	
Ethnicity				
Not Hispanic or Latino	39 (65.00)	22 (70.97)	17 (58.62)	.647
Hispanic or Latino	8 (13.33)	3 (9.68)	5 (17.24)	
Other	8 (13.33)	3 (9.68)	5 (17.24)	
Unknown/not reported	5 (8.33)	3 (9.68)	2 (6.90)	
Marital status				
Married	21 (33.33)	11 (34.38)	10 (32.26)	.593
Single	15 (23.81)	5 (15.63)	10 (32.26)	
Divorced	14 (22.22)	9 (28.13)	5 (16.13)	
Widowed	8 (12.70)	5 (15.63)	3 (9.68)	
Separated	3 (4.76)	1 (3.13)	2 (6.45)	
Living with partner, single	2 (3.17)	1 (3.13)	1 (3.23)	
Lives alone				
No Yes	37 (58.73) 26 (41.27)	21 (65.63) 11 (34.38)	16 (51.61) 15 (48.39)	.259
Highest level of education completed				
Post-doctoral	3 (4.76)	2 (6.25)	1 (3.23)	.144
Doctorate	3 (4.76)	2 (6.25)	1 (3.23)	
Masters level	12 (19.05)	5 (15.63)	7 (22.58)	
University/college	30 (47.62)	18 (56.25)	12 (38.71)	
Technical school	3 (4.76)	2 (6.25)	1 (3.23)	
Associates degree	6 (9.52)	0 (0)	6 (19.35)	
High school	6 (9.52)	3 (9.38)	3 (9.68)	
Previous treatment with mental health professional				
Yes	32 (56.14)	17 (65.38)	15 (48.39)	.198
No	25 (43.86)	9 (34.62)	16 (51.61)	
Primary language				
English	49 (79.03)	22 (70.97)	27 (87.1)	.023^a^
Farsi	6 (9.68)	6 (19.35)	0 (0)	
Spanish	3 (4.84)	2 (6.45)	1 (3.23)	
Other	4 (6.45)	1 (3.23)	3 (9.68)	
Participant medical information				
Actively receiving chemotherapy treatment				
No	20 (37.74)	11 (36.67)	9 (39.13)	.854
Yes	33 (62.26)	19 (63.33)	14 (60.87)	
Actively receiving non-hormonal targeted therapy treatment				
No	9 (30)	4 (28.57)	5 (31.25)	1.000
Yes	21 (70)	10 (71.43)	11 (68.75)	
Actively receiving radiation treatment				
No	51 (96.23)	29 (96.67)	22 (95.65)	1.000
Yes	2 (3.77)	1 (3.33)	1 (4.35)	
Actively receiving hormonal treatment				
No	25 (47.17)	15 (50)	10 (43.48)	.637
Yes	28 (52.83)	15 (50)	13 (56.52)	
History of depression				
No	32 (54.24)	17 (51.52)	15 (57.69)	.636
Yes	27 (45.76)	16 (48.48)	11 (42.31)	
History of anxiety				
No	30 (50.85)	15 (45.45)	15 (57.69)	.351
Yes	29 (49.15)	18 (54.55)	11 (42.31)	
History of bipolar disorder				
No	58 (98.31)	32 (96.97)	26 (100)	1.000
Yes	1 (1.69)	1 (3.03)	0 (0)	
History of other psychiatric disorder				
No	51 (92.73)	27 (87.1)	24 (100)	.123
Yes	4 (7.27)	4 (12.9)	0 (0)	
Location of metastasis				
Bone	29 (51.79)	15 (48.39)	14 (56)	
Brain	3 (5.36)	3 (9.68)	0 (0)	
Liver	9 (16.07)	5 (16.13)	4 (16)	
Other	15 (26.79)	8 (25.81)	7 (28)	
Months since initial diagnosis^*^				
Median (IQR)	49.08 (21.22-111.58)	49.08 (21.78-93.26)	54.19 (15.41-160.97)	.931
Months since initial metastatic disease diagnosis				
Median (IQR)	16.02 (5.49-34.93)	14.98 (5.33-35.36)	16.02 (11.28-29.18)	.515

Data are presented as number of patients (column %) or median (IQR, interquartile range).

*P*-value is calculated by Wilcoxon rank-sum test continuous variables; and chi-square test or Fisher’s exact test for variables, as appropriate.

^a^Indicates statistically significant difference

^*^One patient had a negative value and was not considered.

**Table 2.** Multivariable analyses of spiritual well-being and general quality of life over time.

Variable	Spiritual well-being (FACIT-Spirit-Ex)		General quality of life (FACT-G)	
	Parameter estimate (95% CI)	*P*-value	Parameter estimate (95% CI)	*P*-value
Group effect at each timepoint				
T0	1.76 (−1.58, 5.10)	.297	−2.93 (−6.95, 1.10)	.152
T1	16.67 (13.17, 20.16)	<.001 ^a^	8.51 (4.26, 12.76)	<.001 ^a^
T2	10.31 (6.73, 13.89)	<.001 ^a^	6.17 (1.75, 10.58)	.007 ^a^
Age (years)	0.12 (0.04, 0.2)	.005 ^a^	0.35 (0.24, 0.45)	<.001 ^a^
Race and ethnicity				
Other	^†^		−4.01 (−6.95, −1.07)	.008 ^a^
White			Reference	
Marital status				
Single	^†^		^†^	
Living with partner, single				
Widowed				
Separated				
Divorced				
Married				
Does patient live alone?				
Yes	^†^		^†^	
No				
Highest education (combined)				
Technical school or lower	7.9 (4.97, 10.83)	<.001 ^a^	^†^	
University/college	1.62 (−0.69, 3.93)	.170		
Masters level (18 years)	Reference			
Previous treatment with mental health professional				
Yes	−2.91 (−5.19, −0.63)	.013 ^a^	−7.51 (−10.31, −4.7)	<.001 ^a^
No	Reference		Reference	
Primary language (combined)				
English	^†^		−8.86 (−12.39, −5.34)	<.001 ^a^
Other			Reference	
History of depression				
Yes	−4.58 (−6.7, −2.46)	<.001 ^a^	−8.54 (−11.3, −5.77)	<.001 ^a^
No	Reference		Reference	
History of anxiety				
Yes	^†^		^†^	
No				
History of bipolar disorder				
Yes	^†^		^†^	
No				
History of other psychiatric disorder				
Yes	^†^		^†^	
No				
Months since initial diagnosis^‡^	^†^		^†^	
Location of metastasis				
Brain	^†^		^†^	
Liver				
Other				
Bone				
Months since initial metastatic disease diagnosis	^†^		^†^	

^†^Dropped out of the model.

^‡^One patient had a negative value and was not considered.

^a^Indicates statistically significant difference

### Primary Outcomes

#### Spiritual Well-Being

In univariate analyses, there was no difference in spiritual well-being scores between intervention and control groups at baseline (T0). However, the intervention group had higher spiritual well-being scores at T1 and T2 compared to the control group (Table 2; Fig. 2; Supplementary Table SA). These findings remained the same after adjusting for age, education, previous treatment with mental health professionals, and history of depression (see Table 2).

### Secondary Outcomes

#### General Quality of Life

In univariate analyses, there was no difference in general well-being scores between intervention and control groups at T0. However, the intervention group had higher general well-being scores at T1 and T2 compared to the control group (see Table 2). These findings remained the same after adjusting for age, race/ethnicity, previous treatment with mental health professionals, primary language, and history of depression (see Table 1); (Fig. 2).

#### Anxiety

In univariate analyses, the intervention group had higher anxiety at baseline but tended to have lower anxiety at T1 compared to control. There was no difference in anxiety between the 2 groups at T2 (see Table 3). After adjusting for age, living alone, primary language, and history of depression, the intervention group had higher anxiety at T0 while having lower BAI score at T1 compared to the control group (see Table 3).

**Table 3.** Multivariable analyses of anxiety, depression, hopelessness and loneliness over time.

Variable	Anxiety BAI		Depression BDI-FS		Hopelessness BHS		Loneliness UCLA loneliness v3	
	Parameter estimate (95% CI)	*P*-value	Parameter estimate (95% CI)	*P*-value	Parameter estimate (95% CI)	*P*- value	Parameter estimate (95% CI)	*P*-value
Group effect at each timepoint								
T0	3.26 (1.48, 5.05)	<.001 ^a^	0.42 (−0.29, 1.13)	.245	−0.26 (−1.37, 0.86)	.647	9.09 (6.87, 11.31)	<.001 ^a^
T1	−2.30 (−4.10, −0.51)	.013 ^a^	−1.73 (−2.37, −1.09)	<.001 ^a^	−3.65 (−4.79, −2.51)	<.001 ^a^	6.78 (4.42, 9.14)	<.001 ^a^
T2	−1.10 (−3.04, 0.84)	.264	−1.52 (−2.19, −0.86)	<.001 ^a^	−3.79 (−4.98, −2.61)	<.001 ^a^	2.02 (−0.45, 4.50)	.108
Age at class (years)	−0.17 (−0.21, −0.12)	<.001 ^a^	−0.11 (−0.13, −0.1)	<.001 ^a^	−0.05 (−0.08, −0.03)	<.001 ^a^	−0.15 (−0.21, −0.09)	<.001 ^a^
Race/ethnicity (combined)								
Other	^†^		^†^		^†^		6.67 (5.00, 8.35)	<.001 ^a^
White							Reference	
Marital status								
Single	^†^		^†^		^†^		^†^	
Living with partner, single								
Widowed								
Separated								
Divorced								
Married								
Lives alone								
Yes	−4.6 (−5.77, −3.43)	<.001 ^a^	^†^		^†^		6.4 (4.86, 7.95)	<.001 ^a^
No	Reference						Reference	
Highest education (combined)								
Technical school (14-15 years) or lower	^†^		−1.44 (−2.01, −0.88)	<.001 ^a^	−4.13 (−5.08, −3.19)	<.001 ^a^	^†^	
University/college (15−16 years)			−1.58 (−2.04, −1.11)	<.001 ^a^	−2.34 (−3.1, 1.59)	<.001 ^a^		
Masters level (18 years) or higher			Reference		Reference			
Previous treatment with mental health professional								
Yes	^†^		0.99 (0.6, 1.37)	<.001 ^a^	0.89 (0.12, 1.67)	.025 ^a^	^†^	
No			Reference		Reference			
Primary language (combined)								
English	3.22 (1.85, 4.6)	<.001 ^a^	^†^		^†^		6.78 (5.01, 8.55)	<.001 ^a^
Other	Reference						Reference	
History of depression								
Yes	5.2 (4.12, 6.28)	<.001 ^a^	^†^		1.85 (1.14, 2.56)	<.001^a^	8.31 (6.95, 9.68)	<.001 ^a^
No	Reference				Reference		Reference	
History of anxiety								
Yes	^†^		^†^		^†^		^†^	
No								
History of bipolar disorder								
Yes	^†^		^†^		^†^		^†^	
No								
History of other psychiatric disorder								
Yes	^†^		2.25 (1.49, 3.01)	<.001 ^a^	^†^		^†^	
No			Reference					
Months since initial diagnosis ^‡^								
	^†^		^†^		^†^		^†^	
Location of metastasis								
Brain	^†^		^†^		^†^		^†^	
Liver								
Other								
Bone								
Months since initial metastatic disease diagnosis								
	^†^		^†^		^†^		^†^	

^†^Dropped out of the model.

^‡^One patient had a negative value and was not considered.

^a^Indicates statistically significant difference

#### Depression

In univariate analyses, the intervention group had higher depression at baseline but tended to have lower depression at T1 and T2 compared to control (see Table 3). After adjusting for age, education, previous treatment with a mental health professional, and history of other psychiatric disorders, there was no significant difference in depression between the groups at T0; however, the intervention group had lower depression scores at T1 and T2 (see Table 3).

#### Hopelessness

In univariate analyses, the intervention group seemed to have higher hopelessness scores at T0 while having lower hopelessness at T1 and T2 compared to the control (see Table 3). After adjusting for age, education, previous treatment with a mental health professional, and history of depression, there was no significant difference between the groups at T0; however, the intervention group demonstrated lower hopelessness at T1 and T2 compared to the control (see Table 3).

#### Loneliness

In univariate analyses, the intervention group seemed to have higher loneliness at T0 and T1 and lower loneliness at T2 compared to the control (see Table 3). After adjusting for age, race, live alone, primary language, and history of depression, the intervention group had higher loneliness scores at T0 and T1 compared to control, with no significant difference between the groups at T2 (see Table 3).

## Discussion

The GRACE program was found to provide statistically significant improvements in spiritual well-being and overall quality of life compared to a wait-list control with durability for at least 1 month after the completion of the program. Additional significant improvements were identified for dimensions of general well-being (anxiety, depression, and hopelessness) in multivariate analysis. End of life care is enormously complex.^55,56^ Balancing the dual goals of living as well and as fully as possible while also preparing to face one’s mortality can be extremely challenging.^57,58^ The World Health Organization has underscored the role of palliative care in integrating the psychological and spiritual aspects of patient care and offering a support system to help patients live as actively as possible until death.^59^ The recent Institute of Medicine (IOM) report, *Dying in America* (2015) also highlighted the need for viable programs to address existential and spiritual distress among those with advanced illness.^60^ What is not defined by this landmark report, however, are specifics on *how to pragmatically and effectively address these matters in a real-world clinical setting.*

Traditional support groups are available at many cancer centers and communities around the nation. However, we know from our clinical experience that many of our patients with cancer do not desire to participate in a traditional support group due to a perception that they are unstructured, or they lend themselves to a state of creating comparisons to others in terms of disease state and suffering.^61^ Several studies have concluded that support groups may have little to no benefit^61^ or may harm patients with high levels of support.^62^ Another limitation of many traditional support groups is that they are open-ended and may induce further distress by emphasizing emotions rather than information.^63^

Keeping the limitations of traditional support groups in mind, GRACE was designed to: (1) be finite in duration (ie, 6 sessions) to allow for feasibility on both the part of the participant and institution or clinicians delivering the intervention; and (2) use an integrative psycho-educational process, with Logotherapy as its foundation, focused on enhancing well-being to engage patients with cancer both with high or low levels of psychological distress but still experiencing existential and spiritual challenges. A strength of the GRACE program is the integration of core tenets and techniques from Cognitive Behavioral Therapy, mindfulness intervention, and Positive Psychology to add clinical versatility to complement the foundation of Logotherapy.^29^ GRACE was never intended to create a sense of insincere gratitude about or superficial cognitive framework toward a terrible illness such as metastatic cancer; in contrast, its curriculum was designed with the conviction that the suffering that is commonly experienced in this setting can be mitigated and that skills of resiliency and enhancement of meaning-centered living can be developed and strengthened.

While the intervention demonstrated success in improving spiritual well-being and general quality of life, there are some limitations that must be considered for future research. To begin with, this study was relatively small in its sample size. This may account for the slightly higher anxiety scores at baseline of the intervention group compared to the control group. Secondly, of note, participation in GRACE did not result in improvements in loneliness scores. Loneliness is prominent in this population.^64^ This finding could reflect the length of the intervention (6 sessions) or the nature of the intervention which, while group-based, did not involve interaction of participants outside the intervention. It could also potentially reflect the complexity of loneliness both in terms of optimal measurement and intervention approaches.^65^ However, with knowledge of the linkages between existential distress and loneliness among women with metastatic breast cancer,^64^ it is promising that the primary outcome of distress improved. Future research can explore optimal measures for better assessing loneliness in the intervention or mechanisms for further targeting loneliness through the intervention. Furthermore, future research could include biological markers to gain further insight into the impact of the intervention on objective indicators of distress. Biomarkers shed light on molecular mechanisms of high value in cancer survivorship and allow for relative comparisons of impact of various interventions already established in cancer care, such as yoga, Qi Gong, and aerobic exercise. Biomarkers of merit in cancer biology and wellness may include oxytocin (associated with psychological well-being and positive affective states),^66^ DNA methylation (accelerated aging),^67^ and inflammatory cytokines (known to drive cancer progression and symptom clusters).^68^ Another limitation that can be addressed through future research is that the study did not include an active control group. Participants in the waitlist control did have full access to a well-resourced cancer center, including social work, chaplaincy, and palliative care. However, the utilization of these resources was left up to the participant and not formalized in the control arm. Finally, the target of this research was on women and resiliency, future research can explore the impact of gender on outcomes within this intervention. An additional limitation is that the majority of the participants were White, educated women. This opens the opportunity for adapting the program in a linguistically sensitive way to other communities including a Spanish adaptation of GRACE and a culturally sensitive adaptation targeting historically marginalized oncology populations.

## Conclusion

There exists a critical need to support the well-being and quality of life of women with metastatic cancer, particularly given the high prevalence of existential and spiritual distress in the patient population. The findings of the current randomized clinical trial suggest that the GRACE intervention is successful at improving spiritual well-being, quality of life, and dimensions of general well-being among women with metastatic cancer experiencing existential and spiritual concerns. Future research can explore how to scale the intervention to reach more patients, and how to influence additional key measures of well-being including loneliness.

## Supplementary Material

oyad091_suppl_Supplementary_Table

## Data Availability

The data that support the findings of this study are available from the corresponding author upon reasonable request.
