# Supplementary material for: A 6-Week Program to Strengthen Resiliency Among Women With Metastatic Cancer: A Randomized Clinical Trial
Source: Oncologist. 2023 Apr 27;28(8):e669–82. doi: 10.1093/oncolo/oyad091 (PMC10400157; doi:10.1093/oncolo/oyad091)
Supplement: oyad091_suppl_Supplementary_Table [file oyad091_suppl_supplementary_table.docx]

**Supplemental Table A**

**Table A.** Unadjusted Estimate for Primary and Secondary Outcomes for Each Group over Time

| **Variable** | **Time** | | |
| --- | --- | --- | --- |
|  | **T0** | **T1** | **T2** |
| Spiritual Well-Being (FACIT-Spirit-EX) | | |  |
| Control | 59.54 (57.60, 61.48) | 57.83 (55.63, 60.03) | 59.91 (57.66, 62.16) |
| Intervention | 58.90 (57.02, 60.77) | 71.50 (69.50, 73.50) | 68.44 (66.43, 70.44) |
| Quality of Life (FACT-G) | |  |  |
| Control  Intervention | 60.56 (58.21, 62.92)  57.75 (55.47, 60.03) | 60.59 (57.91, 63.26)  69.30 (66.86, 71.73) | 60.74 (57.95, 63.53)  67.93 (65.41, 70.45) |
| Anxiety (BAI) | | | |
| Control | 18.45 (17.38, 19.53) | 17.59 (16.37, 18.82) | 17.28 (16.04, 18.53) |
| Intervention | 21.92 (20.86, 22.97) | 15.45 (14.33, 16.57) | 16.04 (14.88, 17.20) |
| Depression (BDI) | |  |  |
| Control | 5.79 (5.41, 6.16) | 5.00 (4.58, 5.43) | 5.01 (4.57, 5.44) |
| Intervention | 6.51 (6.14, 6.88) | 3.47 (3.09, 3.86) | 3.54 (3.14, 3.95) |
| Hopelessness (BHS) | |  |  |
| Control | 8.59 (7.90, 9.28) | 8.90 (8.12, 9.68) | 8.68 (7.88, 9.48) |
| Intervention | 9.69 (9.02, 10.37) | 6.17 (5.46, 6.88) | 5.98 (5.23, 6.73) |
| Loneliness (UCLA Loneliness v3) | |  |  |
| Control | 41.07 (39.64, 42.49) | 40.25 (38.63, 41.87) | 42.72 (41.06, 44.37) |
| Intervention | 45.88 (44.50, 47.27) | 42.49 (41.01, 43.96) | 39.71 (38.12, 41.30) |
| Data are presented in estimated score (95% CI) for each group at each timepoint. | | | |
